# Antagonism of LIN-17/Frizzled and LIN-18/Ryk in Nematode Vulva Induction Reveals Evolutionary Alterations in Core Developmental Pathways

**DOI:** 10.1371/journal.pbio.1001110

**Published:** 2011-07-26

**Authors:** Xiaoyue Wang, Ralf J. Sommer

**Affiliations:** Department for Evolutionary Biology, Max-Planck Institut for Developmental Biology, Tübingen, Germany; California Institute of Technology, United States of America

## Abstract

Most diversity in animals and plants results from the modification of already existing structures. Many organ systems, for example, are permanently modified during evolution to create developmental and morphological diversity, but little is known about the evolution of the underlying developmental mechanisms. The theory of developmental systems drift proposes that the development of conserved morphological structures can involve large-scale modifications in their regulatory mechanisms. We test this hypothesis by comparing vulva induction in two genetically tractable nematodes, *Caenorhabditis elegans* and *Pristionchus pacificus*. Previous work indicated that the vulva is induced by epidermal growth factor (EGF)/RAS and WNT signaling in *Caenorhabditis* and *Pristionchus*, respectively. Here, we show that the evolution of vulva induction involves major molecular alterations and that this shift of signaling pathways involves a novel wiring of WNT signaling and the acquisition of novel domains in otherwise conserved receptors in *Pristionchus* vulva induction. First, *Ppa*-LIN-17/Frizzled acts as an antagonist of WNT signaling and suppresses the ligand *Ppa*-EGL-20 by ligand sequestration. Second, *Ppa*-LIN-18/Ryk transmits WNT signaling and requires inhibitory SH3 domain binding motifs, unknown from *Cel*-LIN-18/Ryk. Third, *Ppa*-LIN-18/Ryk signaling involves Axin and β-catenin and *Ppa-axl-1/*Axin is epistatic to *Ppa-lin-18*/Ryk. These results confirm developmental system drift as an important theory for the evolution of organ systems and they highlight the significance of protein modularity in signal transduction and the dynamics of signaling networks.

## Author Summary

Diversity of biological form in animals can be generated by the modification of already existing developmental and morphological structures. One major challenge in evolutionary biology is to identify the molecular and genetic changes associated with such morphological modifications. A decade ago, the theory of developmental systems drift was proposed arguing that large-scale changes in regulatory mechanisms can underlie the development of conserved morphological structures. Our work supports this hypothesis by comparing the development of the egg-laying organ between *Caenorhabditis elegans* and *Pristionchus pacificus*, two nematode species that have significantly different mechanisms of vulva induction despite their morphological similarity. Our studies in *P. pacificus* reveal major molecular alterations of signaling pathways that involve first, a novel wiring and second, the acquisition of novel protein domains in otherwise conserved receptors in WNT signaling. We show that all Wnt signaling molecules analyzed are conserved in sequence, but crucial receptor molecules have acquired novel small peptides that allow new regulatory linkages. The independent evolution of small protein domains in otherwise conserved proteins increases the evolutionary freedom of signaling pathways and developmental networks. Thus, our analysis of a developmental process that follows developmental system drift highlights the significance of protein modularity in signal transduction.

## Introduction

**Figure 1:**
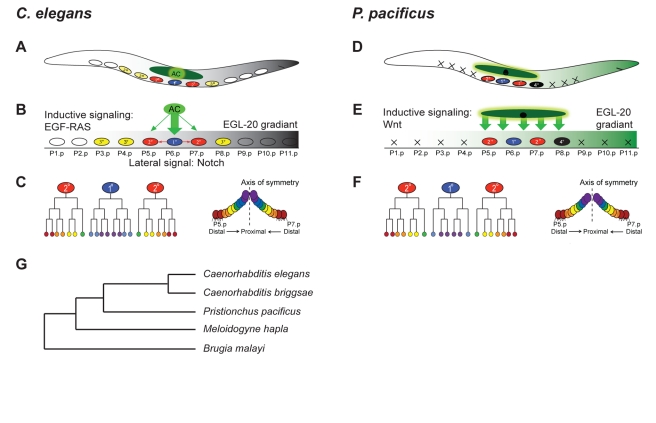
Comparison of cell fate specification in the ventral epidermis in *C. elegans* and *P. pacificus*. (A–C) Vulva formation in *C. elegans*. (A) P(3–8).p form the vulva equivalence group, P(1,2,9–11).p fuse with the hypodermal syncytium *hyp7* (white circles). (B) P(5–7).p adopt a 2°–1°–2° vulval fate pattern (blue and red ovals), while P(3,4,8).p adopt an epidermal fate (3°, yellow ovals). The specification of vulval cell fates depends on LIN-3/EGF signaling, which originates from the gonadal AC (green arrow). P6.p signals its neighbors to adopt a 2° fate via LIN-12/Notch signaling (red arrow). EGL-20/Wnt is expressed as gradient from the posterior tail (grey) and establishes ground polarity of P5.p and P7.p. (C) Cell lineage and cell arrangement of P(5–7).p and their progeny. P6.p divides symmetrically to produce eight progeny that form the inner part of the vulva. P5.p and P7.p divide asymmetrically to produce seven progeny and form the anterior and posterior part of the vulva, respectively. The distal most progeny of P(5,7).p adhere to the epidermis. (D–F) Vulva formation in *P. pacificus*. (D) P(5–7).p adopted a 2°–1°–2° cell fate pattern, P(1–4, 9–11).p die of programmed cell death (black cross). P8.p does not divide but influences the fate of P(5,7).p (4° fate, black oval). (E) *P. pacificus* vulva induction depends on Wnt signaling and Wnt ligands are expressed in the somatic gonad (green arrow) and in the case of *Ppa*-EGL-20 in the posterior tail (green gradient). (F) Cell lineage and cell arrangement of P(5–7).p and their progeny. P6.p divides symmetrically and produces six progeny, whereas P(5,7).p have a cell lineage similar to *C. elegans*. (G) Schematic of the phylogenetic relationship of five nematodes with fully sequenced genomes. The shown distances do not represent the real divergence time for species. For *P. pacificus* and *C. elegans*, a divergence of 250–420 million y has been suggested on the basis of the sequence comparison of more than 1,000 orthologous genes [7].

Biological diversity in metazoans is basically generated by two principles, the evolution of novelty and the modification of already existing structures. Many case studies in recent years have shown that the evolution of novelty often depends on the co-option of developmental control genes in new cells and tissues [1],[2]. However, most diversity in animals and plants is generated by the modification of already existing structures. For example, organ systems are stable over long evolutionary time periods, but they are permanently modified to create a diversity of form and structures [3]. Little is known about the evolution of the molecular mechanisms underlying the specification of such conserved morphological entities. The theory of developmental systems drift proposes that the development of conserved morphological structures can involve large-scale modifications in their regulatory mechanisms [4], but few case studies have been initiated to test this hypothesis. Such studies require detailed functional comparisons between at least two species with the same body plan and they involve the generation of functional approaches in an evo-devo context, i.e., the development of genetic and transgenesis tools in nonclassical model systems [5]. We have developed the nematode *P. pacificus* for such studies allowing detailed analysis of the molecular mechanisms involved in signal transduction. *P. pacificus* belongs to the family of the Diplogastridae, whereas *C. elegans* is a member of the Rhabditidae family [6]. While both groups belong to the same nematode clade, the comparison of more than a thousand orthologous genes suggests that the last common ancestor of *P. pacificus* and *C. elegans* existed 250–420 million y ago (Figure 1G) [7].

To study developmental systems drift we investigate the evolution of vulva induction between *C. elegans* and *P. pacificus*. Both organisms are amenable to forward and reverse genetics and transgenesis, allowing detailed functional and mechanistic studies. *C. elegans* vulva formation serves as one of the major model systems for elucidating the mechanisms of cell fate specification and cell-cell interactions [8]. Therefore, *C. elegans* vulva formation also represents a framework for functional studies in evo-devo [5].

Three of six equipotent *C. elegans* vulval precursor cells (VPCs) are selected by an inductive signal from the gonadal anchor cell (AC) (Figure 1A and 1B) [8]. These three cells, P(5–7).p adopt a 2°–1°–2° fate pattern and form vulval tissue, whereas the three remaining VPCs, P(3,4,8).p, adopt an epidermal, so-called 3° fate (Figure 1B and 1C). A complex network of signaling processes determines vulval cell fates in *C. elegans*. The AC secretes an epidermal growth factor (EGF)-type ligand that is transmitted in the VPCs by RAS/MAPK signaling. A notch-type lateral signaling process acts downstream or in parallel to EGF/RAS signaling to specify vulval fates. Besides EGF and Notch signaling, Wnt signaling has been implicated in two other aspects of vulva determination. First, Wnt signaling is involved in the regulation of the Hox gene *lin-39*, which specifies VPCs early in larval development (Figure S1). In *lin-39*/Hox or *bar-1*/β-catenin mutants, VPCs are not specified and fuse with the hypodermis, adopting the fate of the epidermal cells P(1,2,9–11).p in the anterior and posterior body region (Figure S1) [9]. Later in larval development, another Wnt pathway is involved in the proper execution of the 2° cell lineage (Figure 1C) [10]. While P5.p and P7.p both adopt a 2° cell fate, a lineage reversal of P7.p is required for proper vulva morphogenesis. An atypical Wnt signaling pathway, involving the *Cel-lin-17*/Frizzled, but also the Ryk-type receptor *Cel-lin-18*, was shown to regulate P7.p polarity [10]–[12]. In *Cel-lin-17* and *Cel-lin-18* mutants, the P7.p lineage reversal is abolished and P7.p forms a second vulva-like protrusion in the posterior part of the animal, resulting in a Bivulva phenotype. Thus, Wnt signaling has a key role in the regulation of VPC competence and P7.p polarity in *C. elegans*, whereas its role in vulva induction is still debated [11],[13].

In *P. pacificus*, the vulva is formed by the same cells as in *C. elegans*, P(5–7).p, which also adopt a 2°–1°–2° fate pattern (Figure 1D–1F) [14]. However, vulva induction in *P. pacificus* relies on Wnt signaling and mutants in the Wnt pathway have induction-vulvaless phenotypes, unlike Wnt pathway mutants in *C. elegans* (Figure 1E) [15]. A *Ppa-bar-1/*β–catenin deletion mutant is completely induction-vulvaless. In such animals, VPCs do not divide and remain epidermal (3°), in a manner similar to gonad-ablated animals (Figures S1, S2B, and S2C; Table 1A–1C). Several WNT ligands and receptors act redundantly in *P. pacificus* vulva induction: *Ppa-mom-2*/Wnt; *Ppa-lin-18*/Ryk double mutants and *Ppa-mom-2*; *Ppa-egl-20*; *Ppa-lin-18* triple mutants are strongly vulvaless, while single gene mutations are phenotypically normal (Figure S2D–S2H; Table 1D–1H) [15]. *Ppa-mom-2* and *Ppa-lin-44*, another Wnt ligand, are expressed in the AC and the somatic gonad, respectively, representing the gonadal branch of inductive Wnt signal [15]. In contrast, *Ppa-egl-20* is expressed in the tail in a similar manner as *Cel-egl-20* [15],[16], representing the first signaling ligand involved in vulva induction that is expressed outside of the gonad [15].

**Table 1:** Interactions of Wnt signaling in *P. pacificus* vulva induction.

Experiments	Strains	*n*	Percent WT (3)	Percent Underinduced (<3)	Percent Vulvaless (0)	Percent Overinduced (>3)	Percent P7.p Defects (Biv.)	P(3,4).p Fate	Average induced VPCs
A	WT	100	100	0	0	0	0	PCD	3.0
B	WT Z(1,4)-a	10	0	0	100	0	0	PCD	0.0
C	*Ppa-bar-1(tu362)* a	118	0	1	99	0	0	PCD	0.0
D	*Ppa-egl-20(tu382)* a	50	100	0	0	0	0	PCD	3.0
E	*Ppa-mom-2(tu363)* a	56	83	11	0	0	6	PCD	2.4
F	*Ppa-lin-18(tu359)* a	43	93	5^b^	0	0^c^	2	PCD	2.7
G	*Ppa-mom-2(tu363)*; *Ppa-lin18(tu359)* a	62	2	35	39	0	19	PCD	1.3
H	*Ppa-mom-2(tu363)*; *Ppa-lin18(tu359)*; *Ppa-egl-20(tu364)* a	52	0	40	60	0	0	PCD	0.7
I	*Ppa-lin-17(tu33)* a	50	74	0	0	0	26	PCD	3.0
J	*Ppa-lin-17(tu33)*; *Ppa-egl-20(tu382)* a	49	100	0	0	0	0^d^	PCD	3.0
K	*Ppa-hairy(tu97)* a	50	100	0	0	0	0	3°	3.0
L	*Ppa-ced-3(tu54)*	33	100	0	0	0	0	3°	3.0
M	*Ppa-hairy(tu97)*; *Ppa-lin-17(tu383)*	45	0	0	0	100	0	D	5.9
N	*Ppa-hairy(tu97)*; *Ppa-lin-17(tu383)(Z1,4)-*	10	0	0	0	100	0	D	6.0
O	*Ppa-lin-17(tu383)*	54	3.7	0	0	96.3	0	PCD	4.0
P	*Ppa-hairy(tu97)*; *Ppa-lin-17(tu108)* a	53	0	0	0	47.2	60.4	D	4.4
Q	*Ppa-lin-17(tu33/tu383)*	12	100	0	0	0	0	PCD	3.0
R	*Ppa-lin17(tu383/+)*	58	96.5	0	0	3.4	0	PCD	3.0
S	*Ppa-lin-17(tu383)*; *Ppa-egl-20(tu382)*	50	0	0	0	100	6	PCD	4.0
T	*Ppa-lin-17(tu383)*; *Ppa-lin-18(tu359)*	45	73.3	0	0	13.3^e^	15.6	PCD	3.1
U	*Ppa-axl-1(tu98)*; *Ppa-mom-2(tu263)*; *Ppa-lin-18(tu359)*	22	0	0	0	100^f^	0	PCD	>5
V	*Ppa-lin-17(tu383)*; *Ppa-bar-1(tu362)*	20	0	0	100^g^	0	0	PCD	0.0
W	*Ppa-axl-1(tu98)*; *Ppa-bar-1(tu362)*	29	0	0	100	0	0	PCD	0.0

Worms with wild-type vulva induction have an average number of 3.0 induced VPCs.

a Values originally reported in [15] and [17].

b *p* = 0.2359 (comparison with *Ppa-lin-17(tu383)*; *Ppa-lin-18(tu359)* double mutant vulva underinduction, Fisher exact test).

c *p* = 0.02628 (comparison with *Ppa-lin-17(tu383)*; *Ppa-lin-18(tu359)* double mutant vulva overinduction, Fisher exact test).

d *p*<0.0001 (comparison with *Ppa-lin-17(tu33)* single mutant Bivulva phenotype, Fisher exact test).

e *p*<2.2e–16 (comparison with *Ppa-lin-17(tu383)* vulva Overinduction, Fisher exact test).

f *p*<2.2e–16 (comparison with *Ppa-mom-2(tu363)*; *Ppa-lin-18(tu359)* vulva Overinduction, Fisher exact test).

g *p*<2.2e–16 (comparison with *Ppa-lin-17(tu383)* vulvaless, Fisher exact test).

3°, epidermal cell fate; Biv, Bivulva phenotype; D, ectopic vulva differentiation; PCD, programmed cell death.

Wnt signal transduction during *P. pacificus* vulva induction requires an unusual wiring. In contrast to *Ppa-bar-1*, *Ppa-egl-20*, *Ppa-mom-2*, and *Ppa-lin-18*, which promote vulva induction as indicated by the vulvaless phenotype associated with single or double mutants in these genes [15], *Ppa-lin-17*/Frizzled has a negative role in vulva formation and mutations in *Ppa-lin-17* are multivulva [17]. After ablation of the somatic gonad at hatching, P(5–7).p form vulva-like structures in *Ppa-lin-17(tu33)*. Also, the *Ppa-lin-17* phenotype is suppressed by *Ppa-egl-20*, suggesting a novel ligand-receptor interaction (Figure S2I and S2J; Table 1I and 1J) [15]. A similar interaction between *lin-17* and *egl-20* has subsequently been observed during P7.p polarity specification in *C. elegans* [12]. However, the molecular mechanisms of the *lin-17*–*egl-20* interaction have not been investigated.

Taken together, three hallmarks characterize *P. pacificus* vulva induction when compared to *C. elegans*. First, Wnt signaling rather than EGF/RAS signaling induces the formation of this homologous morphological structure. Second, two signaling centers from the somatic gonad and the posterior body region are redundantly involved in vulva induction relying on distinct Wnt ligands. Third, signaling from the posterior body region involves a novel wiring between the Frizzled receptor *Ppa-lin-17* and the Wnt ligand *Ppa-egl-20*. Here, we elucidate the molecular mechanism of Wnt signaling from the posterior to study developmental systems drift. By using genetic, molecular, and transgenesis studies we show that *Ppa*-LIN-17 acts by ligand sequestration. The Ryk-type receptor *Ppa*-LIN-18 transmits Wnt signaling and requires inhibitory SH3 binding motifs, unknown from *Cel*-LIN-18. *Ppa*-LIN-18/Ryk signaling involves the single Axin-like gene in *P. pacificus* and *Ppa-axl-1* mutants represent the strongest multivulva phenotype observed in *P. pacificus* to date. These studies indicate the acquisition of individual domains in otherwise conserved Frizzled- and Ryk-type receptors during vulva induction in *P. pacificus* and they highlight the strong dynamics and modularity of signaling networks underlying the evolution of conserved morphological structures, thereby supporting the theory of developmental systems drift.

## Results

### Novel Multivulva Mutants with Distinct Vulval Phenotypes

The key difference between *P. pacificus* Wnt signaling during vulva induction and other types of Wnt signal transduction is the antagonistic function of the Frizzled-type receptor *Ppa-lin-17* [15]. While a mutation in the β-catenin–like gene *Ppa-bar-1* is completely vulvaless, *Ppa-lin-17*/Fz null alleles are multivulva as indicated by the ectopic vulva formation in *Ppa-hairy(tu97)*; *Ppa-lin-17/Fz(tu108)* double mutants (Table 1P) [17]. Also, *Ppa-egl-20*/Wnt suppresses the multivulva phenotype of *Ppa-lin-17*/Fz, highlighting the unusual wiring of Wnt signaling during *P. pacificus* vulva induction [15]. To elucidate the molecular mechanism of *Ppa*-LIN-17/Fz function, we used a genetic approach and screened for mutations in genes that would result in a multivulva phenotype similar to *Ppa-lin-17*/Fz. We performed screens in three different genetic backgrounds, wild type, *Ppa-hairy*, and *Ppa-ced-3* mutants. In *Ppa-hairy* and *Ppa-ced-3* mutants, the survival of P(3,4).p results in two epidermal cells that are competent to form vulval tissue (Table 1K and 1L; Figure S2K and S2L) [18],[19]. From these screens we isolated three mutants that fall into two complementation groups. Complementation group 1 has only one allele (*tu383*) and complementation group 2 consists of the alleles *tu98* and *tu329*. We first describe the characterization of *tu383*.

**Figure 2:**
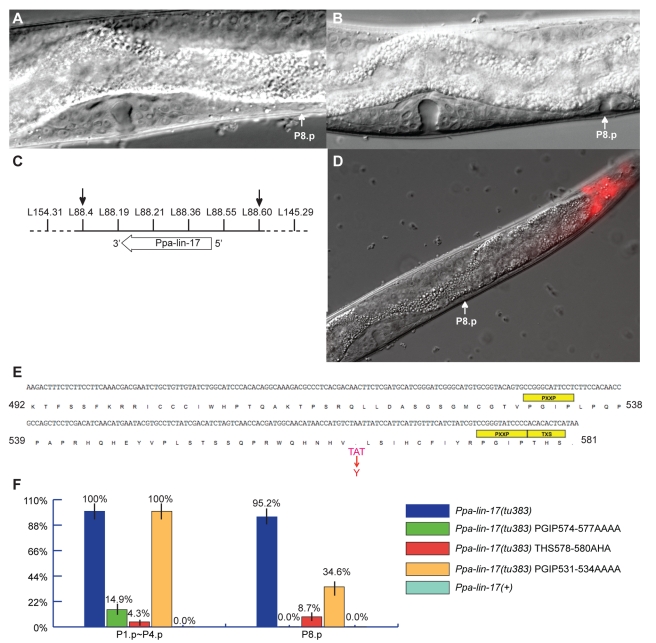
Characterization of *Ppa-lin-17(tu383)* and analysis of its SDBMs. (A) Nomarski photomicrograph of an early J4 larval stage of *P. pacificus* with an undifferentiated P8.p cell (white arrow). (B) Corresponding stage of a *Ppa-lin-17(tu383)* mutant with a vulva-like structure from P8.p (white arrow). (C) Mapping of *tu383*. Markers used for mapping *tu383* to a small region on Chromosome V between markers L88.4 and L88.60 (black arrows). (D) Rescue of *Ppa-lin-17(tu383)*. A 16-kb genomic DNA construct containing a wild-type copy of *Ppa-lin-17* was coinjected with *Ppa-egl-20::*TurboRFP (as marker, red expression in the tail). White arrow points to P8.p, which did not differentiate in transgenic worms. (E) Cloning of *Ppa-lin-17(tu383)*. The intracellular domain of *Ppa-lin-17(tu383)* and conceptual translation are shown. *Ppa-lin-17(tu383)* alters the stop codon (red letters), resulting in a 17 amino acid extension of the protein. The yellow boxes represent the repeated SDBMs, followed by a potential phosphorylation site. (F) Analysis of functional motifs of *Ppa-lin-17(tu383)*. Frequency of vulva induction in P(1–4).p and P8.p were examined in a *Ppa-ced-3(tu54)* mutant background. Transgenic animals carry either a full length *Ppa-lin-17(tu383)* (blue bars), a SDBM-mutated form of *Ppa-lin-17(tu383)* (green, red, and orange bars), or a wild-type *Ppa-lin-17* (light blue bars).

*tu383* has a novel vulva phenotype and was isolated in the *Ppa-hairy* background. In *Ppa-hairy(tu97)*; *tu383* double mutants, P(3–8).p have vulva cell fates and P(3,4,8).p adopt a 2° fate in most mutant animals (Table 1M). After ablation of the somatic gonad at hatching in *Ppa-hairy(tu97)*; *tu383* double mutants, P(3–8).p still adopt vulval cell fates (Table 1N). The *tu383* single mutant retains vulva differentiation in P8.p, whereas P(3,4).p die of programmed cell death (Figure 2A and 2B; Table 1O). These results indicate that *tu383* fulfills both criteria of a multivulva phenotype, ectopic vulva differentiation and gonad-independent vulva formation.

### *tu383* Alters the Stop Codon of *Ppa-lin-17*/Fz and Results in a 17 Amino Acid Extension of the Protein

We mapped *tu383* to a small interval of Chromosome V, which includes *Ppa-lin-17*/Fz (Figure 2C). No other candidate genes related to Wnt signaling are known in this genetic interval. Therefore, we started DNA-mediated transformation rescue experiments by injecting a 16-kb genomic DNA construct that contains the wild-type copy of *Ppa-lin-17*/Fz. More than 50% of transgenic animals showed rescue of the *tu383* phenotype (Figure 2D). We sequenced the *Ppa-lin-17*/Fz gene from *tu383* mutant animals and found an A to T transition changing the natural stop codon TAA to TAT (Figure 2E). This results in a 17 amino acid extension of the *Ppa*-LIN-17/Fz protein. Sequencing of the cDNA by reverse transcriptase (RT)-PCR confirmed this mutation. We conclude that *tu383* represents an unusual allele of *Ppa-lin-17*/Fz.

*Ppa-lin-17*/Fz reduction-of-function alleles and *Ppa-lin-17*/Fz*(tu383)* have distinct vulva phenotypes, affecting P7.p polarity and P8.p differentiation, respectively. P7.p polarity is strongly affected in all *Ppa-lin-17*/Fz reduction-of-function alleles, but not in *Ppa-lin-17*/Fz*(tu383)* (Table 1I and 1O) [17]. By contrast, vulva differentiation in P8.p is only observed in *Ppa-lin-17*/Fz*(tu383)* (Figure 2B). Consistent with these observations, trans-heterozygous *Ppa-lin-17*/Fz*(tu33/tu383)* animals were phenotypically wild type (Table 1Q). Similarly, 56 of 58 *Ppa-lin-17*/Fz(*tu383*/+) animals are phenotypically normal, indicating that the vulva phenotype of *Ppa-lin-17*/Fz*(tu383)* mutants does not have a strong dominant effect (Table 1R). In addition, vulva differentiation of P8.p in *Ppa-lin-17*/Fz*(tu383)* is not suppressed by *Ppa-egl-20*/Wnt (Table 1S). These results indicate that a 17 amino acid extension at the C terminus of *Ppa*-LIN-17/Fz causes a neomorphic phenotype with vulval patterning defects that are distinct from those of *Ppa-lin-17*/Fz reduction-of-function alleles.

### An Ectopic SH3 Domain-binding Motif Is Necessary and Sufficient for the *tu383* Phenotype

Next, we wanted to know if the extension of *Ppa*-LIN-17/Fz in the *tu383* allele changes the biochemical properties of the protein. Interestingly, the 17 amino acid extension contains a potential SH3 domain-binding motif (SDBM) of the sequence PGIP574-577 followed by a potential phosphorylation site THS578-580 (Figure 2E). Another potential SDBM exists in the natural C terminus of *Ppa*-LIN-17/Fz at amino acid position 531–534, which is not followed by a phosphorylation site (Figure 2E).

To test the functional importance of individual amino acids in the C terminus of *Ppa*-LIN-17/Fz*(tu383)*, we developed a transgenic assay with a readout for ectopic vulva differentiation. We used a *Ppa-ced-3(tu54)* mutant background (Figure S2L) and measured ectopic vulva differentiation in both, P(1–4).p and P8.p (Figure 2F). A transgenic line carrying a full length copy of *Ppa-lin-17*/Fz*(tu383)* showed nearly 100% vulva differentiation in P(1–4).p and P8.p (Figure 2F, dark blue bars). In contrast, the transgenic line containing a wild-type copy of *Ppa-lin-17*/Fz did not show ectopic vulva differentiation in these cells (Figure 2F, light blue bars).

Using this assay system, we tested the function of the potential SDBM sites in the intracellular domain of *Ppa-*LIN-17/Fz*(tu383)*. First, we generated a transgene with a PGIP574-577AAAA mutation. Transgenic animals showed a reduction in P(1–4).p vulva differentiation from 100% to 15% and vulva differentiation in P8.p is completely abolished (Figure 2F, green bars). Second, we generated a construct that changed THS578–580 into AHA. Animals carrying this transgene showed vulva differentiation below 10% in P(1–4).p and P8.p (Figure 2F, red bars). In contrast, transgenic animals with a mutation of PGIP531–534 to AAAA showed 100% vulval differentiation in P(1–4).p and 35% in P8.p (Figure 2F, orange bars). These experiments suggest that the multivulva phenotype of *Ppa-lin-17*/Fz*(tu383)* depends on the PGIPTHS motif in the 17 amino acid extension of the *Ppa*-LIN-17/Fz receptor. We speculate that this protein extension provides an ectopic SDBM that interferes with other protein interactions in VPCs. One potential mechanism would be that PGIPTHS574-580 mimics a peptide of another protein in the Wnt signaling network, i.e., the receptor for *Ppa-egl-20*/Wnt, and competes for binding to a regulatory protein that acts downstream in the Wnt pathway.

### The Intracellular Domain of *Ppa*-LIN-18/Ryk Is Required for Vulva Induction

**Figure 3:**
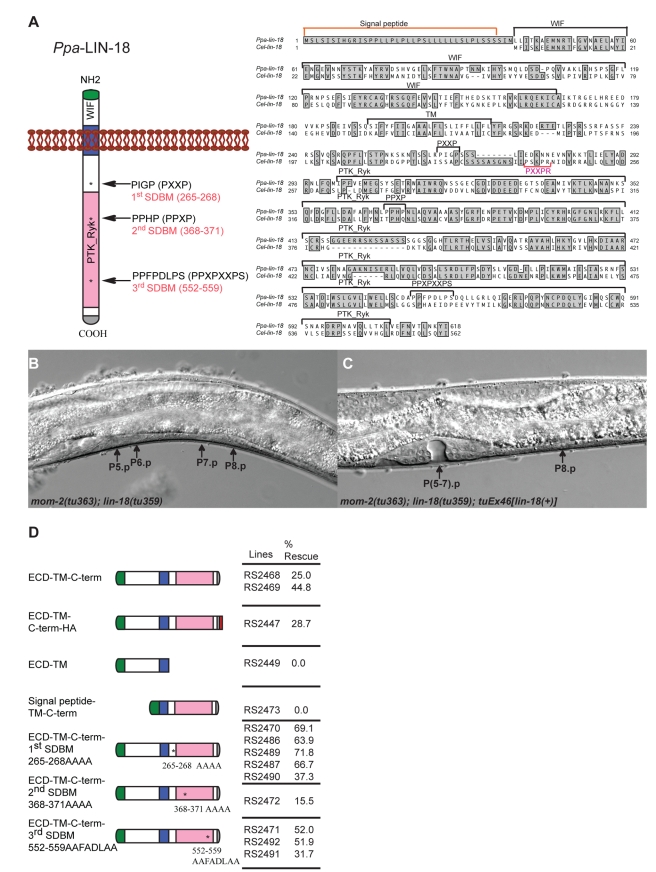
Molecular structure and function of *Ppa-lin-18* and analysis of SDBMs in *Ppa*-LIN-18. (A) Left, *Ppa*-LIN-18 protein structure. Wnt-inhibitory factor-1 like domain (WIF domain, white), Receptor related to tyrosine kinase (PTK_Ryk, pink) and transmembrane domain (TM, blue). Positions of three potential SDBMs of *Ppa*-LIN-18 are indicated by arrows. Right, amino acid sequence alignment of *Ppa*-LIN-18 and *Cel*-LIN-18. The positions of SDBMs in *Ppa*-LIN-18 are shown by black letters, the single potential SDBM in *Cel*-LIN-18 is shown in pink. (B) Nomarski photomicrograph of a J4 larval stage of a *Ppa-mom-2(tu63)*; *Ppa-lin-18(tu359)* double mutant with a vulvaless phenotype. Black arrows point to the undifferentiated VPCs. (C) Corresponding stage of a transgenic animal carrying a wild-type copy of *Ppa*-LIN-18. P(5–7).p differentiated and formed normal vulva, P8.p remains epidermal (black arrows). (D) Rescue efficiency of *Ppa-mom-2(tu363)*; *Ppa-lin-18(tu359)* by different *Ppa*-LIN-18 constructs (signal peptide, green; WIF domain, white; TM domain, blue; PTK_Ryk domain, pink). The WT-LIN-18-HA construct contains an HA epitope tag, “MYPYDVPDYA” (red) in front of the stop codon of *Ppa*-LIN-18.

Next, we searched for potential SDBMs in other *P. pacificus* Frizzled-type receptors and *Ppa*-LIN-18/Ryk. While none of the other *P. pacificus* Frizzled-type receptors contains an SDBM, there are three such motifs in the intracellular domain of *Ppa*-LIN-18 (Figure 3A), the Ryk-type receptor that is known to be involved in vulva induction (Figure S2F–S2H; Table 1F–1H) [15]. Sequence comparison between *Ppa*-LIN-18/Ryk and *Cel*-LIN-18/ryk reveals high sequence similarity in both, the extracellular and intracellular parts of the LIN-18/Ryk proteins (Figure 3A, right). The intracellular domain consists of a kinase domain, which in *C. elegans* is not required for P7.p polarity [10]. While the *P. pacificus* and *C. elegans* LIN-18/Ryk proteins show a high overall sequence similarity, *Ppa*-LIN-18/Ryk contains three potential SDBMs, whereas only one sequence with weak similarity to this motif is found in *Cel*-LIN-18/Ryk (Figure 3A, right).

To study the function of individual domains of *Ppa*-LIN-18/Ryk, we developed a transgenic assay with a functional reporter system. In *Ppa-lin-18*/Ryk; *Ppa-mom-2*/Wnt double mutants only 2% of the worms form a normal vulva with a 2°–1°–2° pattern, whereas 39% of the animals are vulvaless (Figures 3B and S2G; Table 1G). Transgenic animals that carry a full-length wild-type copy of *Ppa-lin-18*/Ryk can partially rescue the *Ppa-lin-18*/Ryk; *Ppa-mom-2*/Wnt double mutant phenotype. In two independent transgenic lines, 25% and 45% of animals form a normal vulva (Figures 3C, 3D, and S3). Similarly, a full-length *Ppa*-LIN-18/Ryk fused to an HA tag at the carboxyl terminus showed 29% rescue of the double mutant phenotype (Figures 3D and S3). Next, we generated constructs containing only the extracellular domain and the transmembrane domain (“ECD-TM” construct), or the signal peptide, the transmembrane domain and the intracellular domain (“Signal peptide-TM-C-term” construct). None of these transgenic lines rescued the *Ppa-lin-18*/Ryk; *Ppa-mom-2*/Wnt double mutant phenotype, suggesting that in contrast to *C. elegans*, the extracellular domain of *Ppa*-LIN-18/Ryk on its own does not have rescuing activity (Figures 3D and S3). These results provide the first evidence that *Ppa*-LIN-18/Ryk requires C-terminal domain in vulva induction and has a distinct function than in *C. elegans*.

### *Ppa*-LIN-18/Ryk Contains Inhibitory SH3 Domain-binding Motifs

To determine if the three potential SDBMs in the intracellular domain of *Ppa*-LIN-18/Ryk are involved in the regulation of Wnt signaling, we mutated the SDBM encoding fragments of *Ppa-lin-18*/Ryk and tested for rescuing activity of the resulting transgenes. Transgenic lines carrying mutations of the first or third SDBM (“ECD-TM-C-term 1st SDBM265-268AAAA” and “ECD-TM-C-term 3rd SDBM552-559AAFDLAA”) showed a strong increase in rescuing activity (Figures 3D and S3). Specifically, four of five transgenic lines with mutations at SDBM265-268 showed more than 64% rescue. Similarly, two out of three lines with mutations at SDBM552-559 showed more than 50% rescue (Figures 3D and S3). In contrast, mutations of the second site, SDBM368-371, resulted in normal vulval patterning in only 15% of transgenic animals (Figures 3D and S3). These results suggest that SDBM sites 1 and 3 of *Ppa*-LIN-18/Ryk have an inhibitory function, which might be involved in binding to an inhibitor that prevents signaling in the absence of Wnt ligands. We speculate that the removal of SDBMs 1 or 3 in *Ppa*-LIN-18/Ryk results in an enhancement of rescuing activity, because in the absence of these SDBMs the inhibitor can no longer bind to *Ppa*-LIN-18/Ryk. Under the assumption that the ectopic SDBM site PGIPTHS574-580 of *Ppa*-LIN-17/Fz*(tu383)* mimics the SDBMs of *Ppa*-LIN-18/Ryk, *Ppa*-LIN-17/Fz*(tu383)* attracts the inhibitor by competitive binding from *Ppa*-LIN-18/Ryk, thereby causing ligand independent signal transduction.

### The SH3 Domain-binding Motifs of *Ppa*-LIN-18/Ryk and *Ppa*-LIN-17/Fz*(tu383)* Have Similar Binding Properties

**Figure 4:**
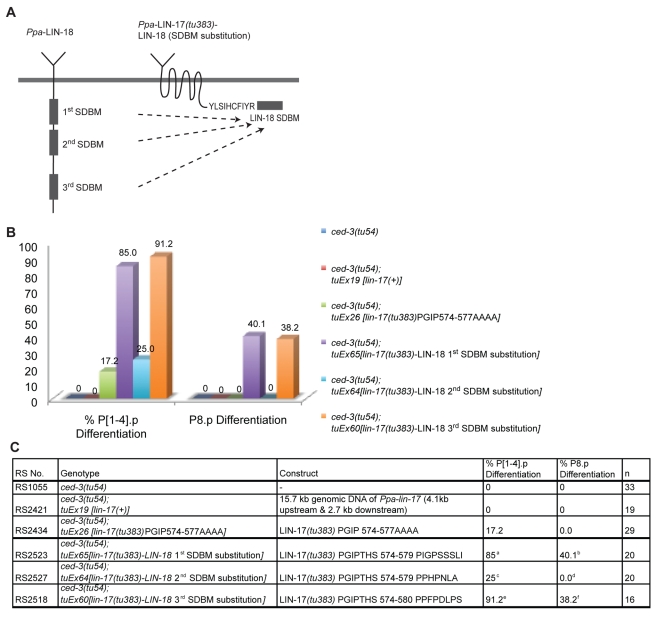
Chimeric *Ppa*-LIN-*17(tu383)*-LIN-18 (SDBM) transgenes cause ectopic vulva formation. (A) Design of chimeric constructs of *Ppa*-LIN-17*(tu383)*-LIN-18 (SDBM). The ectopic SDBM site of *Ppa*-LIN-17*(tu383)* was substituted by the SDBMs of *Ppa*-LIN-18 one by one. (B, C) Graphical (B) and numerical (C) analysis of vulva induction after overexpression of chimeric constructs in a *Ppa-ced-3(tu54)* background. Experimental design is similar to experiments in Figure 3F. Purple and orange bars show vulva differentiation in transgenic animals carrying chimeric constructs of *Ppa*-LIN-17*(tu383)* substitutions with the first and third SDBM of *Ppa*-LIN-18. In contrast, substitution of *Ppa*-LIN-17*(tu383)* with the second SDBM of *Ppa*-LIN-18 substitution showed limited vulva differentiation in P(1–4).p and P8.p. Statistic analysis: ^a^ *p*-value = 4.575e–06; ^b^ *p*-value = 0.0003; ^c^ *p*-value = 0.72; ^d^ *p*-value = 1; ^e^ *p*-value = 6.069e–07; ^f^ *p*-value = 0.001 (comparison with *ced-3(tu54)*; *tuEx26 [lin-17(tu383)*PGIP574-577AAAA], Fisher exact test).

The experiments described above indicate that the SDBMs are important for *Ppa*-LIN-18/Ryk function and they are consistent with the hypothesis that *Ppa*-LIN-18/Ryk and *Ppa*-LIN-17/Fz*(tu383)* competitively bind inhibitory SH3 domain proteins via their SDBMs. To study if *Ppa*-LIN-18/Ryk and *Ppa*-LIN-17/Fz*(tu383)* have similar binding properties, we replaced the ectopic SDBM PGIPTHS574-580 of *Ppa*-LIN-17/Fz*(tu383)* with the individual SDBMs of *Ppa*-LIN-18/Ryk (Figure 4A) and tested the resulting constructs in the transgenic assays for ectopic vulva differentiation in P(1–4).p and P8.p using the *Ppa-ced-3(tu54)* mutant background (Figure 4). Transgenic animals carrying construct in which the ectopic SDBM of *Ppa*-LIN-17/Fz*(tu383)* was replaced by the first or third SDBM of *Ppa*-LIN-18/Ryk, showed strong vulva induction in P(1–4).p and P8.p (Figure 4B and 4C). In contrast, transgenic animals carrying a *Ppa*-LIN-17/Fz*(tu383)* substitution with the second SDBM of *Ppa*-LIN-18/Ryk, showed only 25% vulva induction in P(1–4).p, which is similar to the result of transgenes containing a *Ppa*-LIN-17/Fz*(tu383)* PGIP574-577AAAA mutation (Figure 4B and 4C). These results indicate that the ectopic SDBM of *Ppa*-LIN-17/Fz*(tu383)* mimics the first and third SDBM sites of *Ppa*-LIN-18/Ryk, suggesting that *Ppa*-LIN-17/Fz*(tu383)* and *Ppa*-LIN-18/Ryk competitively bind to a common SH3 domain containing inhibitory protein.

If *Ppa*-LIN-18/Ryk and *Ppa*-LIN-17/Fz*(tu383)* compete for binding, a *Ppa-lin-18*/Ryk*(tu359)* mutant should be epistatic to *Ppa-lin-17*/Fz*(tu383)*. Indeed, *Ppa-lin-18*/Ryk*(tu359)* suppresses the multivulva phenotype of *Ppa-lin-17*/Fz*(tu383)* and 33 of 45 (73%) *Ppa-lin-18*/Ryk*(tu359)*; *Ppa-lin-17*/Fz*(tu383)* double mutants are phenotypically wild type (Table 1T), indicating that *Ppa-lin-17*/Fz*(tu383)* requires functional *Ppa*-LIN-18/Ryk for signaling. Taken together, these experiments suggest that the SDBMs in *Ppa*-LIN-18/Ryk and *Ppa*-LIN-17/Fz*(tu383)* have similar SH3 binding properties and that *Ppa-lin-18*/Ryk*(tu359)* suppresses *Ppa-lin-17*/Fz*(tu383)*.

### *tu98* and *tu329* Represent the *Pristionchus axin* Gene

**Figure 5:**
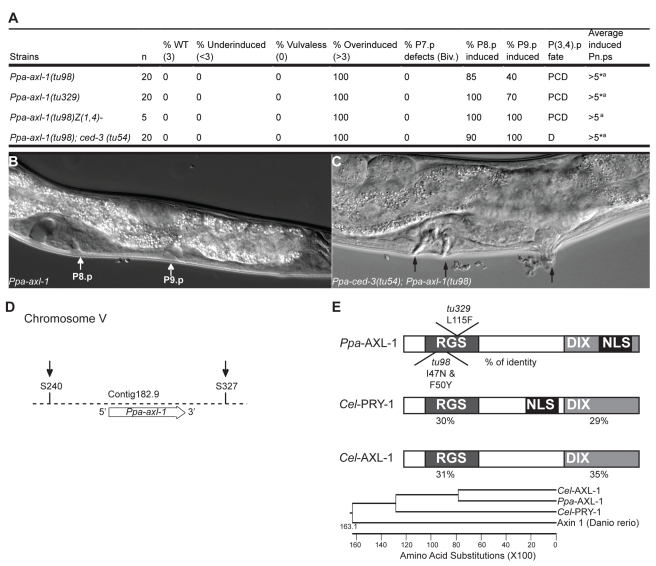
Molecular cloning and characterization of *Ppa-axl-1*. (A) Vulval patterning defects of *tu98* and *tu329* alleles. Besides P(5–7).p, P8.p and often the surviving posterior cells P(9–11).p form vulva-like structures. *Average number of Pn.p cells with vulva-like differentiation is above 5. We did not count differentiation of P(10,11).p in the posterior region as it is known that these cells also form vulva-like protrusions in a *ced-3* mutant background. (B) Nomarski photomicrograph of an early J4 larval stage of a *Ppa-axl-1(tu98)* mutant animal with a vulva-like structure in P8.p and P9.p (white arrows). (C) Nomarski photomicrograph of a late J4 larval stage of a *Ppa-axl-1(tu98);Ppa-ced-3(tu54)* double mutant with ectopic vulva-like structures in the anterior and posterior body region (black arrows). (D) Mapping of *tu98* and *tu329* to an interval on Chromosome V between markers S240 and S327 (black arrows). (E) Domain structure and position of mutations in *Ppa-axl-1*. *Ppa*-AXL-1 is most similar in sequence to *Cel*-AXL-1 and contains a RGS and the “Dishevelled-Axin” (DIX) domain. Mutations in both alleles are in the RGS domain. *tu98* was induced by trimethylpsoralen/UV light treatment and contains two mutations, a phenomenon known to occur after this type of treatment. Phylogenetic tree of AXIN encoding genes. Branch length is proportional to the number of substitutions per site.

Next, we characterized the remaining multivulva locus (complementation group 2, see above), which represents the strongest multivulva phenotype obtained in *P. pacificus* to date. Two alleles, *tu98* and *tu329*, have been isolated from large-scale mutagenesis experiments. *tu98* and *tu329* mutant animals have two ventral epidermal cell lineage defects, both of which indicate that this gene represents a negative regulator of vulva formation. First, P8.p adopts a vulva fate in 85% and 100% in *tu98* and *tu329* mutant animals, respectively (Figure 5A and 5B). Second, P(9–11).p survive and adopt vulval cell fate in most mutant animals (Figure 5A and 5B). Furthermore, P(5–11).p can adopt vulval fates after ablation of the somatic gonad at hatching, indicating that the multivulva phenotype is gonad independent (Figure 5A). Similarly, in a *Ppa-ced-3(tu54)*; *tu98* double mutant all epidermal cells from P1.p to P11.p can undergo vulva differentiation (Figure 5A and 5C).

We mapped the locus to an interval of several hundred kb between the molecular markers S240 and S327 in the center of Chromosome V (Figure 5D). One potential candidate in that region is a gene with high sequence similarity to *axin*, a known negative regulator of Wnt signaling in *C. elegans* [20],[21]. While *C. elegans* contains two *axin*-like genes, *Cel-pry-1* and *Cel-axl-1*, the current draft of the *P. pacificus* genome has only one *axin*-like gene, which is called *Ppa-axl-1* because it shows highest sequence similarity to *Cel-axl-1* (Figure 5E). Overall, sequence similarity is restricted to the known functional domains, the “Regulator of G Protein Signaling” (RGS) and the “Dishevelled-Axin” (DIX) domain (Figure 5E).

To determine if this complementation group corresponds to *Ppa-axl-1*, we performed rescue experiments. A 7-kb genomic construct containing the complete coding region as well as 2.8 kb of upstream sequence fully rescued the phenotype of *tu98*. When we sequenced the *Ppa-axl-1* gene in the two alleles *tu98* and *tu329*, we found mutations in the RGS domain in both of them. *tu329* contains a mutation resulting in an amino acid alteration from Leu to Phe at position 115 and the trimethylpsoralen-ultraviolet–induced allele *tu98* contains two mutations in the RGS domain resulting in amino acid alterations at position 47 (Ile to Asn) and position 50 (Phe to Tyr) (Figure 5E). All mutations were confirmed by sequencing cDNA fragments. Together, these results indicate that loss-of-function alleles in *Ppa-axl-1* result in the strongest known multivulva phenotype in *P. pacificus*.

### *Ppa-axl-1* Is Epistatic to *Ppa-lin-18*/Ryk

Next, we studied the genetic interactions between *Ppa-lin-18*/Ryk, *Ppa-axl-1*, and *Ppa-bar-1*/β-catenin. First, we generated a *Ppa-axl-1(tu98)*; *Ppa-mom-2*/Wnt*(tu263)*; *Ppa-lin-18*/Ryk*(tu359)* triple mutant to see if *Ppa-axl-1(tu98)* can suppress the vulvaless phenotype of *Ppa-mom-2*/Wnt*(tu263)*; *Ppa-lin-18*/Ryk*(tu359)* double mutants. Indeed, a *Ppa-axl-1(tu98)*; *Ppa-mom-2*/Wnt*(tu263)*; *Ppa-lin-18*/Ryk*(tu359)* triple mutant is multivulva indicating that *Ppa-axl-1* is epistatic to *Ppa-lin-18*/Ryk (Table 1U). Next, we determined the role of *Ppa-bar-1*/ß-catenin in relation to the other factors. *Ppa-axl-1(tu98)*; *Ppa-bar-1*/β-catenin*(tu362)* and *Ppa-bar-1*/ß-catenin *(tu362)*; *Ppa-lin-17*/Fz*(tu383)* double mutants are completely vulvaless, indicating that the unusual Wnt network acting during *P. pacificus* vulva induction is *Ppa*-BAR-1/β-catenin dependent, with *Ppa*-BAR-1/ß-catenin representing the most downstream component identified to date (Table 1V and 1W).

### *Ppa*-EGL-20/Wnt Overexpression Induces Ectopic Vulva Formation

**Figure 6:**
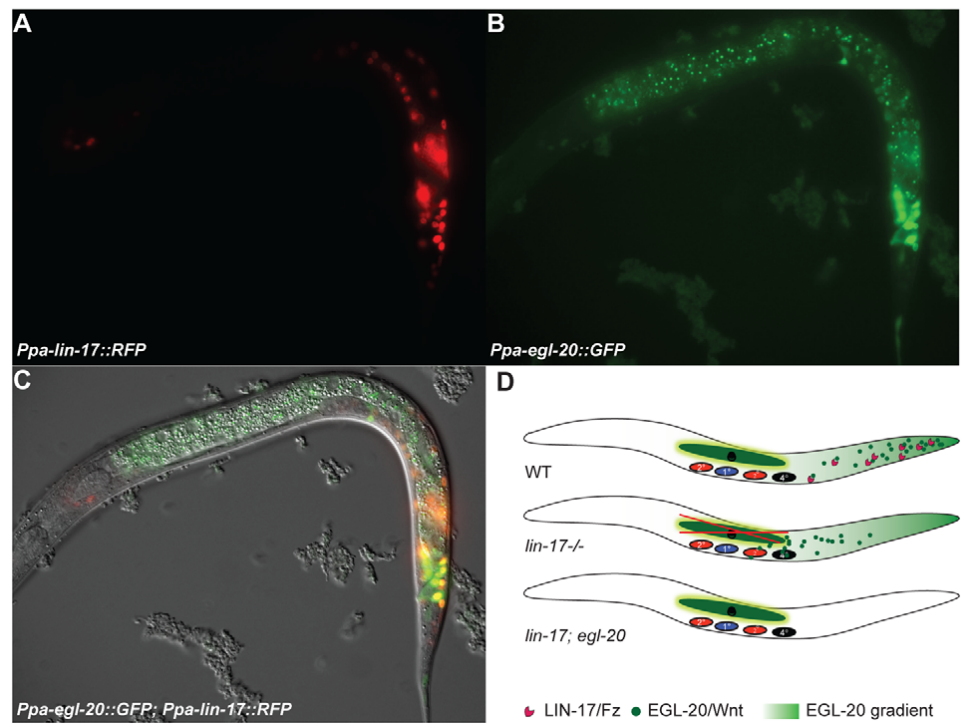
Expression of *Ppa-lin-17* and *Ppa-egl-20* control vulva induction in *P. pacificus*. (A–C) J2 larval stage of wild-type *P. pacificus* animal. (A) *Ppa-lin-17* promoter driving nuclear localized TurboRFP expression in the posterior tail. (B) Within the same animal, GFP under the control of the *Ppa-egl-20* promoter. (C) Merged image of *Ppa-egl-20*::GFP; *Ppa-lin-17*::TurboRFP. (D) Ligand sequestration model. In wild type, *Ppa*-LIN-17 sequesters *Ppa*-EGL-20 in the posterior body region. In *Ppa-lin-17* mutant animals, excessive *Ppa*-EGL-20 ligand reaches the central body region induces vulva formation in the absence of the signal from the second signaling center, the somatic gonad. A mutation in *Ppa-egl-20* fully suppresses the gonad-independent vulva differentiation phenotype of *Ppa-lin-17*.

The experiments described above indicate an antagonism of *Ppa*-LIN-17/Fz and *Ppa*-LIN-18/Ryk during *Ppa*-EGL-20/Wnt signaling from the posterior body region. Two testable predictions derive from these observations. First, the antagonistic role and the multivulva phenotype of *Ppa-lin-17*/Fz mutants suggest that *Ppa*-LIN-17/Fz works by ligand sequestration of *Ppa*-EGL-20/Wnt. According to this model, *Ppa*-LIN-17/Fz balances the amount of *Ppa*-EGL-20/Wnt from the posterior body region. In a *Ppa-lin-17*/Fz null mutant ectopic *Ppa*-EGL-20/Wnt signal will reach the VPCs causing vulva formation in the absence of the signal from the second signaling center, the somatic gonad (Figure 6D). Second, *Ppa-lin-17*/Fz should be coexpressed with *Ppa-egl-20*/Wnt in the posterior body region.

To test the ligand sequestration model (Figure 6D), we expressed *Ppa-egl-20*/Wnt under two constitutive promoters and determined if *Ppa-egl-20*/Wnt overexpression was sufficient to cause ectopic vulva formation. We expressed *Ppa-egl-20*/Wnt under the control of the *Drosophila* heat shock promoter *Dm*-hsp-70 and the *Ppa-mig-1*/Fz promoter, respectively, and studied the effect of *Ppa*-EGL-20/Wnt overexpression in *Ppa-ced-3*; *Ppa-egl-20*/Wnt double mutant animals. The *Ppa-egl-20*/Wnt background eliminates endogenous Wnt activity and the cell-death defective *Ppa-ced-3* background results in the survival of P(1–4,9–11).p (Figure S2L). We analyzed ectopic vulva differentiation in P(1–4).p, the cells furthest away from the endogenous source of *Ppa*-EGL-20/Wnt signaling in the posterior body region. In *Dm*-hsp-70*::Ppa-egl-20*/Wnt transgenic animals, vulva differentiation in P(1–4).p was on average observed in 25% of heat shocked animals, whereas *Ppa-mig-1::Ppa-egl-20*/Wnt transgenic animals showed 12% vulva differentiation in these cells (Table 2). In contrast, nontransgenic *Ppa-ced-3; Ppa-egl-20*/Wnt mutant animals have no ectopic vulva differentiation (Table 2). Thus, *Ppa-egl-20*/Wnt is sufficient for vulva induction, supporting the ligand sequestration model for *Ppa*-LIN-17/Fz (Figure 6D).

**Table 2:** *Ppa*-EGL-20/WNT overexpression induces ectopic vulva formation.

Genotype	Heat Shock Experiment	Percent P[1–4].p Differentiation	*n*
*Ppa-ced-3(tu104)*; *Ppa-egl-20(tu382)*; *tuEx33[Dm-hsp70::*EGL-20*]*	1	38.5	13
	2	30.8	13
	3	22.2	36
	4	23.0	13
	5	16.1	31
	6	10.3	29
	7	33.3	9
	Average	24.9*	N.A.
*Ppa-ced-3(tu104)*; *Ppa-egl-20(tu382)*; *tuEx35[Ppa-mig-1::*EGL-20*]*	N.A.	11.8**	51
*Ppa-ced-3(tu104)*; *Ppa-egl-20(tu382)*	N.A.	0.0	32

Staged *Ppa-ced-3(tu104)*; *Ppa-egl-20(tu382)* worms (19 h after hatching), containing *tuEx33[Dm-hsp70::EGL-20]* transgene were heat shocked for 80 min at 35°C. Late J2 and early J3 larval stage were scored 18 h later.

*p = 0.0004;

**p = 0.0773 (comparison with Ppa-ced-3(tu104); Ppa-egl-20(tu382), Fisher exact test).

N.A., not available.

### *Ppa-lin-17*/Fz Is Expressed in the Posterior Body Region

To study the expression pattern of *Ppa-lin-17*/Fz we generated a *Ppa-lin-17*/Fz*::RFP(NLS)* construct containing 1.5-kb upstream sequence of *Ppa-lin-17*/Fz. We found *Ppa-lin-17*/Fz expression in the posterior body region surrounding the rectal area in all larval stages (Figure 6A–6C). Coexpression of *Ppa-lin-17*/Fz::RFP with *Ppa-egl-20*/Wnt::GFP indicates that the two genes have an overlapping expression pattern during larval development that is, however, not completely identical (Figure 6A–6C). Low level *Ppa-lin-17*/Fz::RFP expression is also seen in more anterior regions including the VPCs during the J3 larval stage (unpublished data). All described expression patterns were highly reproducible and were observed in several independent transgenic lines. This pattern is consistent with ligand sequestration and the antagonistic interaction of *Ppa*-EGL-20/Wnt and *Ppa*-LIN-17/Fz (Figure 6D). However, given the *Ppa-lin-17*/Fz expression in the posterior body region and the VPCs, we cannot distinguish if ligand sequestration occurs in the posterior body region, the central body region, or both. To address this question, we expressed *Ppa-lin-17*/Fz under the *Ppa-egl-20/*Wnt promoter. A *Ppa-egl-20/*Wnt::*Ppa-lin-17*/Fz transgene that is only expressed in the posterior region showed rescue of the *Ppa-lin-17*/Fz*(tu108)* multivulva phenotype in a *Ppa-ced-3(tu104)* mutant background (Figure S4). These experiments suggest that ligand sequestration is largely restricted to the posterior tail region.

## Discussion

Transcription factors and signaling pathways are highly conserved throughout the animal kingdom [22],[23]. Nonetheless, morphological and developmental structures show a near endless diversity. How this diversity is generated in the context of conserved developmental control genes remains largely elusive. We try to tackle this problem by studying the evolution of the developmental mechanisms underlying the specification of a conserved developmental and morphological structure, the nematode vulva. While previous studies clearly indicated that the vulva is a conserved organ that is formed from homologous precursor cells, little was known about the exact molecular mechanisms at work [24]. Here, we determined the molecular mechanisms of Wnt signaling from the posterior body region during *P. pacificus* vulva induction and demonstrate the importance of modularity and dynamics of signaling networks for the evolution of developmental mechanisms.

We have obtained evidence for a novel wiring in a Wnt signaling network with a ligand sequestration function of LIN-17/Fz and the coupling of the LIN-18/Ryk receptor to Axin and BAR-1/β-catenin regulation. Four major conclusions can be drawn from these results. First, differences in vulva induction between *C. elegans* and *P. pacificus* are not restricted to the differential use of EGF/RAS versus Wnt signaling. The shift of signaling pathways involves a novel wiring of Wnt signaling. Second, signaling pathways that are conserved throughout the animal kingdom are highly dynamic and alterations of individual protein interactions can be under strong influence of small protein domains. Therefore, the presence or absence of SDBMs in nematode LIN-18/Ryk receptors highlights the significance of protein modularity for evolution. Also, evolutionary conserved proteins differ between species in that LIN-17/Fz acts as antagonist in *P. pacificus* vulva induction but as agonist in most Wnt signaling pathways. This supports a third conclusion, that mutational changes in protein-coding regions of core developmental control genes do occur and are of evolutionary importance. The significance of mutations in coding regions that change the structure of proteins versus mutations in *cis*-regulatory elements that alter the expression pattern of a given gene has been the subject of a recent debate in evo-devo [25],[26]. Several case studies provide evidence for both types of mutation patterns, but these studies centered on adaptive changes between closely related species [27],[28]. The comparison of vulva induction between *C. elegans* and *P. pacificus* provides an example for the evolutionary mutation spectrum of core developmental pathways. Because both nematodes are amenable to forward and reverse genetics, functional and mechanistic studies are possible that made it feasible to identify functional changes in the coding region of developmental control genes [5]. Our study therefore, provides support for the claim that the discussed prevalence of mutations in *cis*-regulatory elements might result from an ascertainment bias [27].

Finally, the comparison of Wnt signaling in animal development reveals a diversity of molecular mechanisms that is to a certain extent independent of phylogeny. For example, the coupling of the LIN-18/Ryk receptor to β-catenin regulation in *P. pacificus* is more similar to what has been described in mammals than to *C. elegans* or *Drosophila* Ryk function [29]–[31]. Similar findings were made previously when studying the role of GROUCHO proteins and their interaction with basic helix-loop-helix transcription factors in *P. pacificus* and *C. elegans* [19].

**Figure 7:**
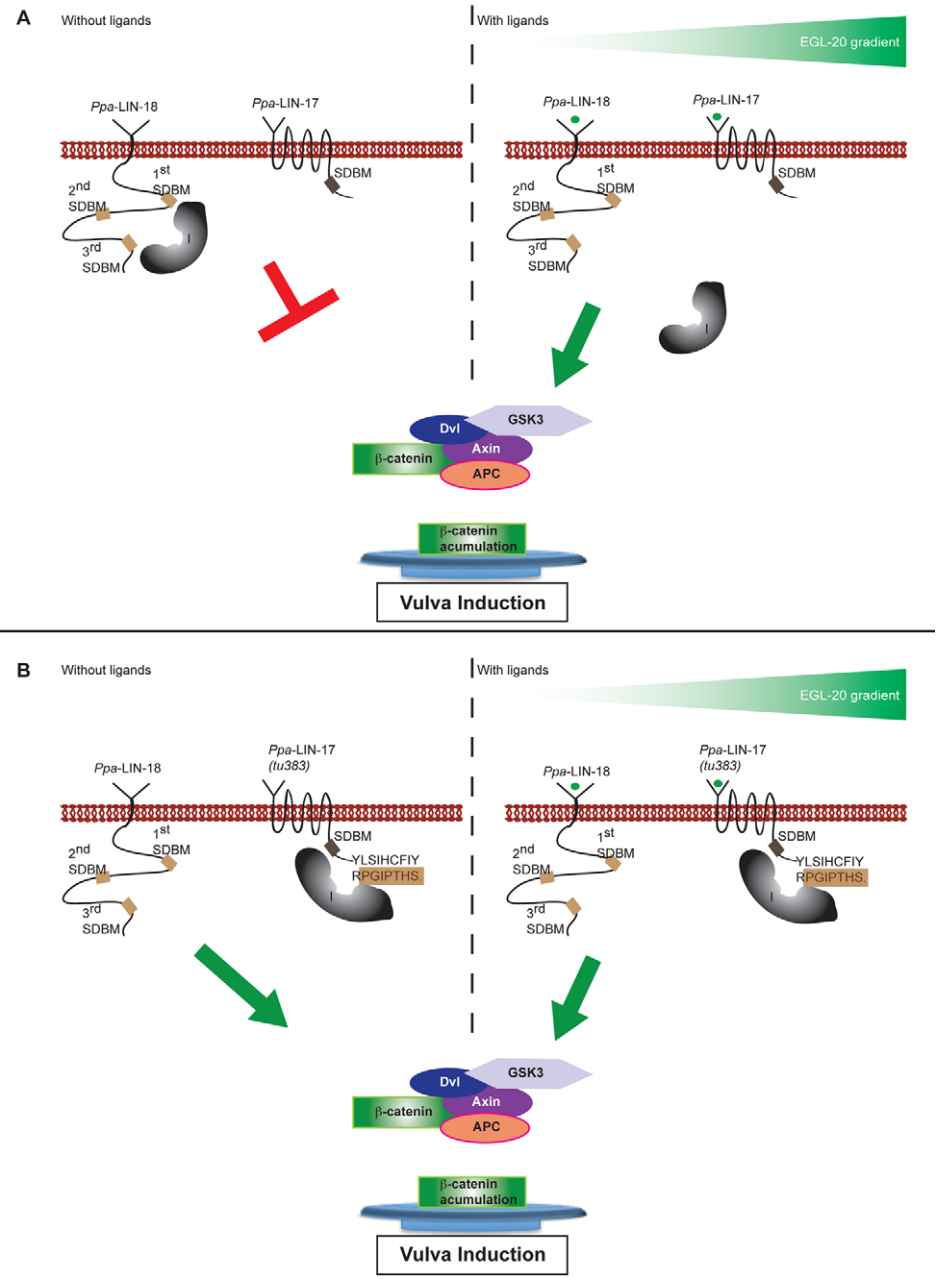
Model for the antagonism of *Ppa*-LIN-17 and *Ppa*-LIN-18 during *Ppa*-EGL-20 signaling. (A) Vulva induction in wild type. Left, the first and third SDBM sites in *Ppa*-LIN-18 bind to an inhibitor (I), which in the absence of Wnt ligands prevents signal transduction. Right, *Ppa*-EGL-20 acts as one of the inducers of *P. pacificus* vulva formation. When the amount of *Ppa*-EGL-20 outcompetes ligand sequestration by *Ppa*-LIN-17, the inhibitor is released from *Ppa*-LIN-18 and signaling occurs. This *Ppa*-LIN-18/Ryk signaling involves Axin and β-catenin. (B) Vulva induction in *Ppa-lin-17(tu383)* mutant conditions. Left, the ectopic SDBM site of *Ppa-lin-17(tu383)* attracts the inhibitor and causes the release from *Ppa*-LIN-18, resulting in ligand-independent vulva differentiation. Consistently, the *Ppa-lin-17(tu383)* mutant phenotype is not suppressed by *Ppa-egl-20* mutants.

In this study we report what to our knowledge are three novelties of Wnt signaling in nematode development. First, we have discovered a new mechanism for LIN-17/Fz function in Wnt signaling. We hypothesize that *Ppa*-LIN-17/Fz balances the amount of *Ppa*-EGL-20/Wnt leading to ligand sequestration (Figures 6D and 7A). In a *Ppa-lin-17*/Fz mutant, *Ppa*-EGL-20/Wnt is not properly sequestered resulting in gonad-independent vulva differentiation and a multivulva phenotype (Figure 6D). Ligand sequestration is most likely restricted to the posterior body region close to the source of *Ppa*-EGL-20/Wnt ligand, as suggested by the observed rescuing capability of a *Ppa-egl-20*/Wnt-driven *Ppa-lin-17*/Fz transgene in a *Ppa-lin-17(tu108)*; *Ppa-ced-3(tu104)* background (Figure S4). In *C. elegans*, a related regulation of EGL-20/Wnt activity was described by the Ror receptor tyrosine kinase CAM-1 [32]. *Cel*-EGL-20/Wnt regulates the anterior migration of the hermaphrodite-specific neuron (HSN) and the right Q neuroblast. Overexpression of *Cel-egl-20*/Wnt, as well as *cam-1* mutants result in too far anterior migration, indicating that *Cel*-CAM-1 negatively regulates *Cel*-EGL-20/Wnt. In *Drosophila*, the Frizzled-type receptor *Dfz2* has also been reported to antagonize Wnt signaling [33].

Second, the analysis of the neomorphic *Ppa-lin-17*/Fz*(tu383)* allele provides insight into a novel aspect of the molecular mechanism of Wnt signaling. The mutation of the natural stop codon and the resulting 17 amino acid extension in *Ppa*-LIN-17/Fz*(tu383)* led to the identification of a potential SDBM and three similar SDBMs in *Ppa*-LIN-18/Ryk. We propose that *Ppa*-EGL-20/Wnt signaling is transduced by *Ppa*-LIN-18/Ryk and the SDBM sites in *Ppa*-LIN-18/Ryk act as negative regulators (Figure 7A). Through the binding of an inhibitor or an inhibitory complex, these SDBM sites prevent ligand-independent Wnt signal transduction. In the neomorphic *Ppa-lin-17*/Fz*(tu383)* allele, the inhibitor is attracted by the ectopic SDBM site in *Ppa*-LIN-17/Fz*(tu383)* and the release of the inhibitor from *Ppa*-LIN-18/Ryk results in ligand-independent vulva differentiation and a multivulva phenotype (Figure 7B). This model is supported by the fact that *Ppa-lin-18*/Ryk*(tu359)* suppresses the multivulva phenotype of *Ppa-lin-17*/Fz*(tu383)*. However, the effect of *tu383* and its 17 amino acid extension is specific to *Ppa-lin-17*/Fz as indicated by the finding that the fusion of the ectopic SDBM site to another Fz-type receptor, *Ppa*-MIG-1/Fz, does not result in a similar multivulva phenotype as *Ppa-lin-17*/Fz*(tu383)* (unpublished data).

To our knowledge, this study provides the first evidence for the involvement of SDBMs in Wnt signaling. We speculate that the inhibitor or inhibitory complex in *P. pacificus* Wnt signaling involves SH3 domain proteins, the molecular nature of which awaits future analysis. There are 38 SH3 domain proteins in the *P. pacificus* genome including representatives of all major genes known from other model organisms (Table S1) [34]. Given the fact that the mutagenesis screens for vulva defective mutants in *P. pacificus* have been saturated and no uncloned multivulva locus remains, it is well possible that redundancy masks the function of individual genes in this process. It is interesting to note that in contrast to Wnt signaling, SH3 domain proteins are known to play a key role in EGF/RAS signaling. In *C. elegans* vulva induction, the GRB2 homolog *sem-5* is a positive regulator of vulva formation [35], whereas *sli-1*, a member of the Cbl ubiquitin ligase family, is a negative regulator of LET-23/EGFR [36]. One intriguing possibility therefore would be that *Ppa*-LIN-18/Ryk interacts with any of these gene products.

Third, the functional characterization of *Ppa-axl-1* shows that the novel wiring of *Ppa*-EGL-20/Wnt, *Ppa*-LIN-17/Fz, and *Ppa*-LIN-18/Ryk requires Axin and acts through the β-catenin–type protein *Ppa*-BAR-1. No in vivo genetic evidence has been described previously between LIN-18/Ryk, Axin, and β-catenin. Data from *C. elegans* and mammalian cell culture provide evidence that Ryk is required for TCF-driven transcription [37],[38]. Similarly, a recent study described the isolation of a *pry-1*/Axin mutation in *C. briggsae* [39]. While *Cbr-pry-1*/Axin mutants provide evidence for the conservation and diversification of Wnt signaling in *Caenorhabditis*, *Cbr-pry-1*/Axin was shown to function upstream of *Cbr-bar-1|*β-catenin and *Cbr-pop-1* [39].

Taken together, the comparison of Wnt pathway components and their functions between *P. pacificus* and *C. elegans* reveals an enormous diversity in the regulatory linkage of Wnt signal transduction and in the composition of the regulatory networks that control conserved morphological entities. Therefore, the evolution of vulva development provides a prime example for developmental systems drift [4]. The results described in this study highlight the strong dynamics and modularity of signaling networks in animal development and can therefore be used to extend the concept of developmental systems drift further. The evolution of individual protein domains, and not necessarily complete genes or proteins, can result in novel protein interactions and thus, new regulatory linkages. These findings result in the intriguing idea that protein domains might be the prime subjects of natural selection. This idea would be consistent with the general notion that developmental control genes are highly conserved in sequence throughout the animal kingdom. This conservation however, is often restricted to the DNA binding domains of transcription factors or already well-characterized domains of intracellular proteins. Small peptides, like the SDBM described here, can and most likely, do evolve relatively fast. However, functionally important peptides might often remain unnoticed, as they cannot be simply deduced from computational analysis. Only a detailed genetic and biochemical characterization can identify the functional significance of such domains. The independent evolution of small protein domains in otherwise conserved proteins increases the “evolutionary freedom” of signaling pathways and developmental networks. The introduction of a new interaction partner into an already existing pathway, but also the cross-connectivity of signaling pathways in developmental networks can be facilitated by the evolution of novel protein domains. Thus, the acquisition of novel protein domains might allow the de novo evolution of functional modules in the context of conserved regulatory control genes.

## Materials and Methods

### Nematode Work

All worms were kept on nematode growth medium (NGM) agar plates with the *Escherichia coli* strain OP50 as food [14]. *P. pacificus* PS312 (California) was used for all genetic analysis. For cell ablation experiments, animals were picked into M9 buffer placed on a pad of 5% agar in water containing 10 mM sodium azide as anesthetic. All ablation experiments were carried out 0–1 h after hatching (20°C).

### Genetics

The following mutants were used: *Ppa-bar-1*/β-catenin*(tu362)*, *Ppa-egl-20*/Wnt*(tu382)*, *Ppa-mom-2*/Wnt *(tu363)*, *Ppa-lin-18*/Ryk*(tu359)*, *Ppa-mom-2*/Wnt *(tu363)*; *Ppa-lin-18*/Ryk*(tu359)*, *Ppa-mom-2*/Wnt *(tu363)*; *Ppa-lin-18*/Ryk*(tu359)*; *Ppa-egl-20*/Wnt *(tu364)*, *Ppa-lin-17*/Fz*(tu33)*; *Ppa-egl-20*/Wnt *(tu382)* (all originally described in [15]), *Ppa-hairy(tu97)* [19], *Ppa-lin-17*/Fz*(tu33)* [17]. The following single and double mutants are newly described in this study: *Ppa-lin-17*/Fz*(tu383)*, *Ppa-hairy(tu97)*; *Ppa-lin-17*/Fz *(tu383)*, *Ppa-lin-17*/Fz *(tu383/tu33)*, *Ppa-lin-17*/Fz *(tu383)*; *Ppa-egl-20*/Wnt*(tu382)*, *Ppa-lin-17*/Fz *(tu383)*; *Ppa-lin-18*/Ryk*(tu359)*, *Ppa-axl-1(tu98)*; *Ppa-mom-2*/Wnt*(tu363)*; *Ppa-lin-18*/Ryk*(tu359)*, *Ppa-lin-17*/Fz *(tu383)*; *Ppa-bar-1*/β-catenin*(tu362)*, *Ppa-axl-1(tu98)*; *Ppa-bar-1*/β-catenin *(tu362)*.

### Mutagenesis Screens

Mixed stage animals were washed off the plates in M9 buffer, after which ethyl methanesulphonate (EMS) was added to a final concentration of 50 mM for 4 h at 20°C. After washing in M9 five times, worms were spotted onto the surface of NG plates. After 1 h, excess liquid had been absorbed and individual motile L4 hermaphrodites were picked individually to plates. In the F2 generation, egg-laying defective mutants were isolated and their progeny were reanalyzed for vulva defects using Nomarski microscopy.

### Mapping and SSCP Detection

For genetic mapping, mutant hermaphrodites in the California background were crossed with males of the Washington strain [40]. To extract genomic DNA, F2 mutant animals were picked into single tubes each containing 2.5 µl of lysis buffer (50 mM KCl; 10 mM Tris-HCl [pH 8.3]; 2.5 mM MgCl_2_; 0.45% NP-40; 0.45% Tween; 0.01% gelatin; 5 µg/ml proteinase K) and incubated for 1 h at 65°C, followed by inactivation of the proteinase K at 95°C for 10 min. To assign linkage of a mutation to a certain chromosome, two representative SSCP markers per chromosome were tested against 42 Washington-backcrossed mutant animals. For SSCP detection, PCR samples were diluted 1∶1 in denaturing solution (95% formamide, 0.1% xylene cyanol, 0.1% bromophenol blue), denatured at 95°C for 5 min, and loaded onto a GeneGel Excel prepoured 6% acrylamide gel (PharmaciaBiotech).

### Transformation

Extrachromosomal arrays were generated by coinjecting a transgene with the marker *Ppa-egl-20*/Wnt: TurboRFP (10 ng/µl) and genomic carrier DNA (60 ng/µl) into wild-type *P. pacificus*, *Ppa-lin-17*/Fz*(tu383)*, *ced-3(tu54)*, *ced-3(tu54)*; *egl-20*/Wnt*(tu382)*, or *Ppa-lin-18*/Ryk*(tu359)*; *Ppa-mom-2*/Wnt*(tu363)* hermaphrodites as described previously [41]. Extrachromosomal arrays for rescue of *Ppa-lin-17*/Fz*(tu383)* were made by injecting 5 ng/µl of a *Ppa-lin-17*/Fz wild-type construct into *Ppa-lin-17*/Fz*(tu383)* mutants. Rescue of *tu98* and *tu329* was made by injecting 0.5 ng/µl of a 7-kb *Ppa-axl-1* PCR construct together with *tu98* genomic carrier DNA. *Ppa-egl-20*:TurboRFP expression was used to identify transgenic animals. For all constructs, multiple independent lines were generated. Expression constructs showed highly reproducible expression patterns between lines and animals. A total list of transgenes used in this study is provided as Table S2.

## Supporting Information

Figure S1 **Distinct functions of Wnt signaling during** ***P. pacificus*** **and** ***C. elegans*** **vulva formation.** In *C. elegans* (to the left), *bar-1/*β-catenin and *lin-39*/HOX mutants show a similar phenotype, which is distinct from gonad-ablated animals. In contrast, *Ppa-bar-1*/β-catenin mutant animals show a phenotype that is distinct from *Ppa-lin-39*/HOX mutant, but is similar to the phenotype of gonad-ablated animals, indicating that Wnt signaling plays a dominant role in *Pristionchus* vulva induction.
(TIF)

Figure S2 **Illustration of vulval cell fates and phenotype in different mutant backgrounds.** This figure represents a graphical presentation of the data shown in Table 1. The different vulval cell fates are indicated by red circles (2° cell fate), blue circles (1° cell fate), yellow circles (3° cell fate). The black circle indicates the 4° nonvulval cell fate. Black cross indicates programmed cell death. In “I,” the brown figure on top of P7.p, indicates the phenotype of polarity defect. The percentage of phenotypes is indicated in “G”, “H,” and “J.”
(TIF)

Figure S3 **Rescue of** ***Ppa-mom-2(tu363)*** **;** ***Ppa-lin-18(tu359)*** **by** ***Ppa-lin-18*** **transgenes.** Detailed description of the data summarized in Figure 3D. Late J2 or early J3 stage worms were observed. The upper part provides a description of the transgenes used in this analysis; the lower part indicates the percentage of animals with a wild-type phenotype. ^#^ *p*-values were calculated by comparisons with *Ppa-mom-2(tu363)*; *Ppalin-18(tu359)* in percentage of wild-type animals, Fisher exact test.
(TIF)

Figure S4 **Rescue of** ***Ppa-lin-17(tu108)*** **multivulva phenotype by expressing LIN-17 in the tail region.** (A) Late J2 or early J3 stage worms were observed. Worms with wild-type vulva induction have an average number of 3.0 induced VPCs. Statistical analysis: a *p*-value = 7.451e–06 [comparison with *Ppa-lin-17(tu108)*; *Ppa-ced-3(tu104)* P(3,4).p vulva induction, Fisher exact test]. b *p*-value = 0.005066 [comparison with *Ppa-lin-17(tu108)*; *Ppa-ced-3(tu104)* P8.p vulva induction]. c *p*-value = 3.883e–05 [comparison with *Ppa-lin-17(tu108)*; *Ppa-ced-3(tu104)* P9.p vulva induction]. (B) The percentage of vulva induction in P(3,4).p, P8.p, and P9.p. The blue bars represent the percentage of vulva induction in *Ppa-lin-17(tu108)*; *Ppa-ced-3(tu104)* double mutant, and the red bars represent the percentage of vulva induction in *Ppa-lin-17(108)*; *Ppa-ced-3(tu104)*; *tuEX99[egl-20::lin-17cDNA]* transgenic animals.
(TIF)

Table S1 ***P. pacificus*** **gene predictions for genes encoding SH3 domain containing proteins.**
(PDF)

Table S2 **Composition of the constructs used to generate transgenic lines described in this study.**
(PDF)

## Abbreviations

AC: anchor cell
EGF: epidermal growth factor
RGS: Regulator of G Protein Signaling
SDBM: SH3 domain-binding motif
VPC: vulval precursor cell
