# Supplementary figures and images for: Antagonism of LIN-17/Frizzled and LIN-18/Ryk in Nematode Vulva Induction Reveals Evolutionary Alterations in Core Developmental Pathways
Source: PLoS Biol. 2011 Jul 26;9(7):e1001110. doi: 10.1371/journal.pbio.1001110 (PMC3144188; doi:10.1371/journal.pbio.1001110)

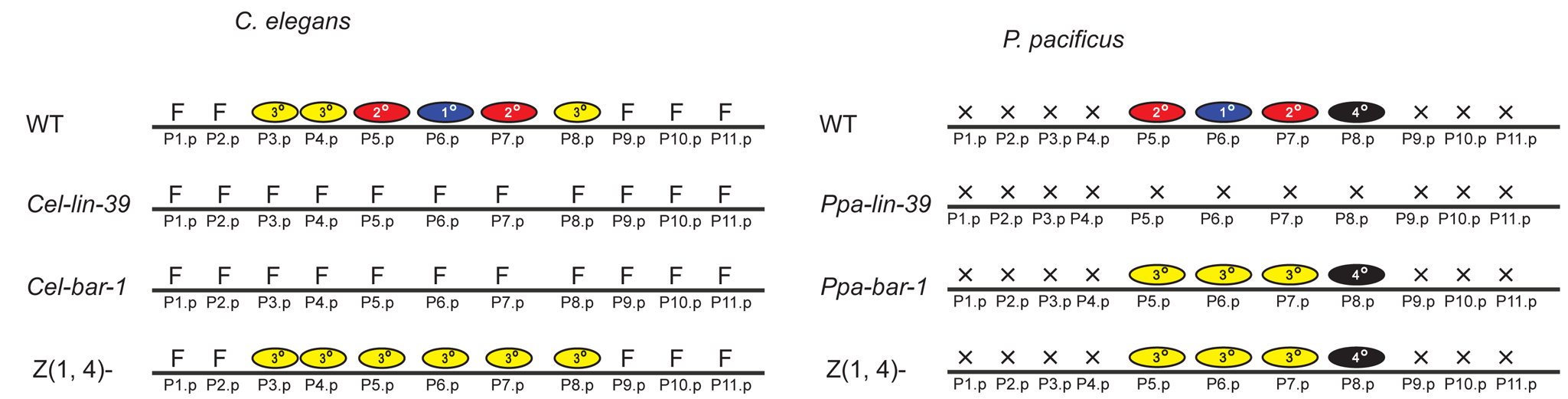

Supplement: Figure S1 — Distinct functions of Wnt signaling during P. pacificus and C. elegans vulva formation. In C. elegans (to the left), bar-1/β-catenin and lin-39/HOX mutants show a similar phenotype, which is distinct from gonad-ablated animals. In contrast, Ppa-bar-1/β-catenin mutant animals show a phenotype that is distinct from Ppa-lin-39/HOX mutant, but is similar to the phenotype of gonad-ablated animals, indicating that Wnt signaling plays a dominant role in Pristionchus vulva induction. (TIF) [file pbio.1001110.s001.tif]

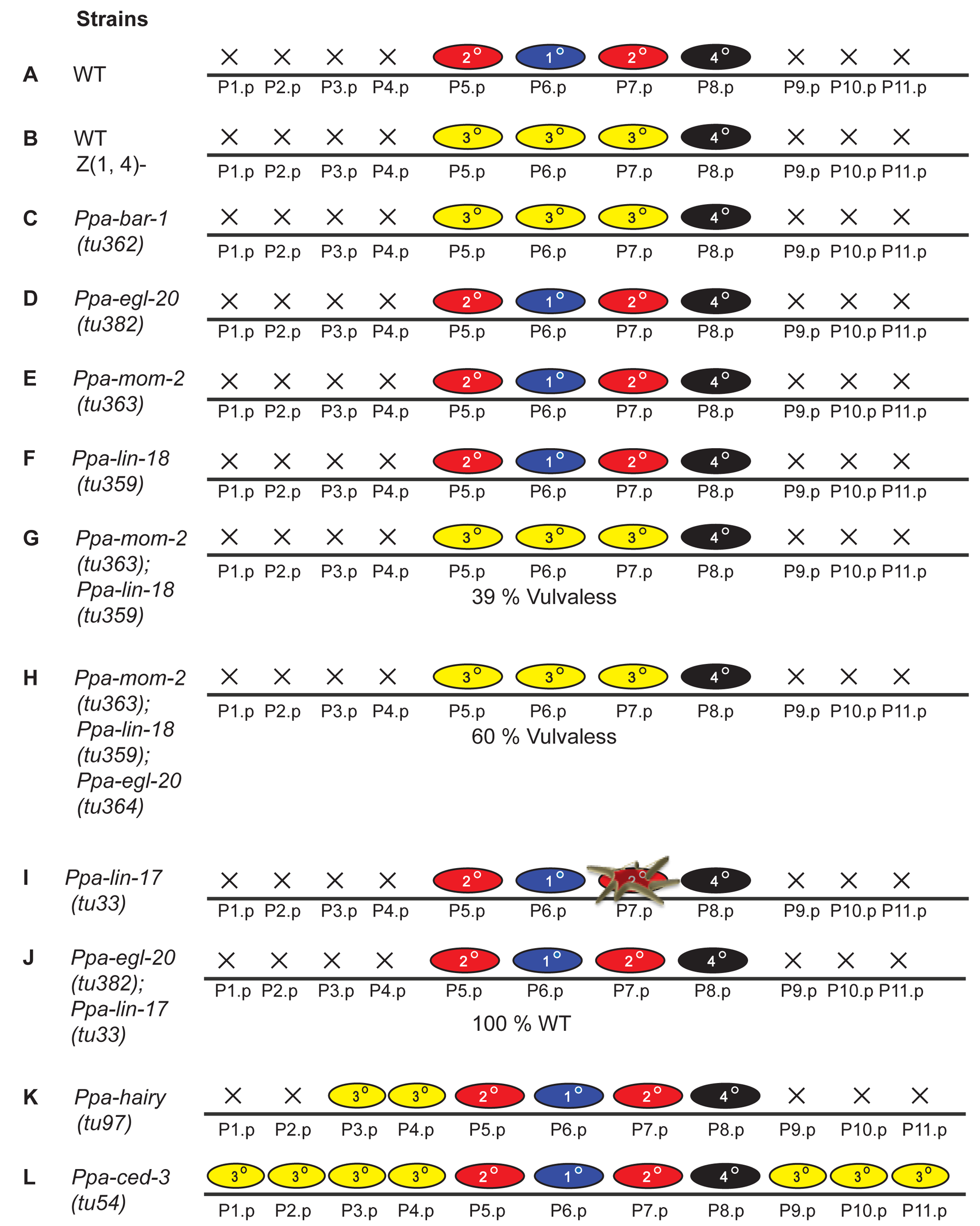

Supplement: Figure S2 — Illustration of vulval cell fates and phenotype in different mutant backgrounds. This figure represents a graphical presentation of the data shown in Table 1. The different vulval cell fates are indicated by red circles (2° cell fate), blue circles (1° cell fate), yellow circles (3° cell fate). The black circle indicates the 4° nonvulval cell fate. Black cross indicates programmed cell death. In “I,” the brown figure on top of P7.p, indicates the phenotype of polarity defect. The percentage of phenotypes is indicated in “G”, “H,” and “J.” (TIF) [file pbio.1001110.s002.tif]

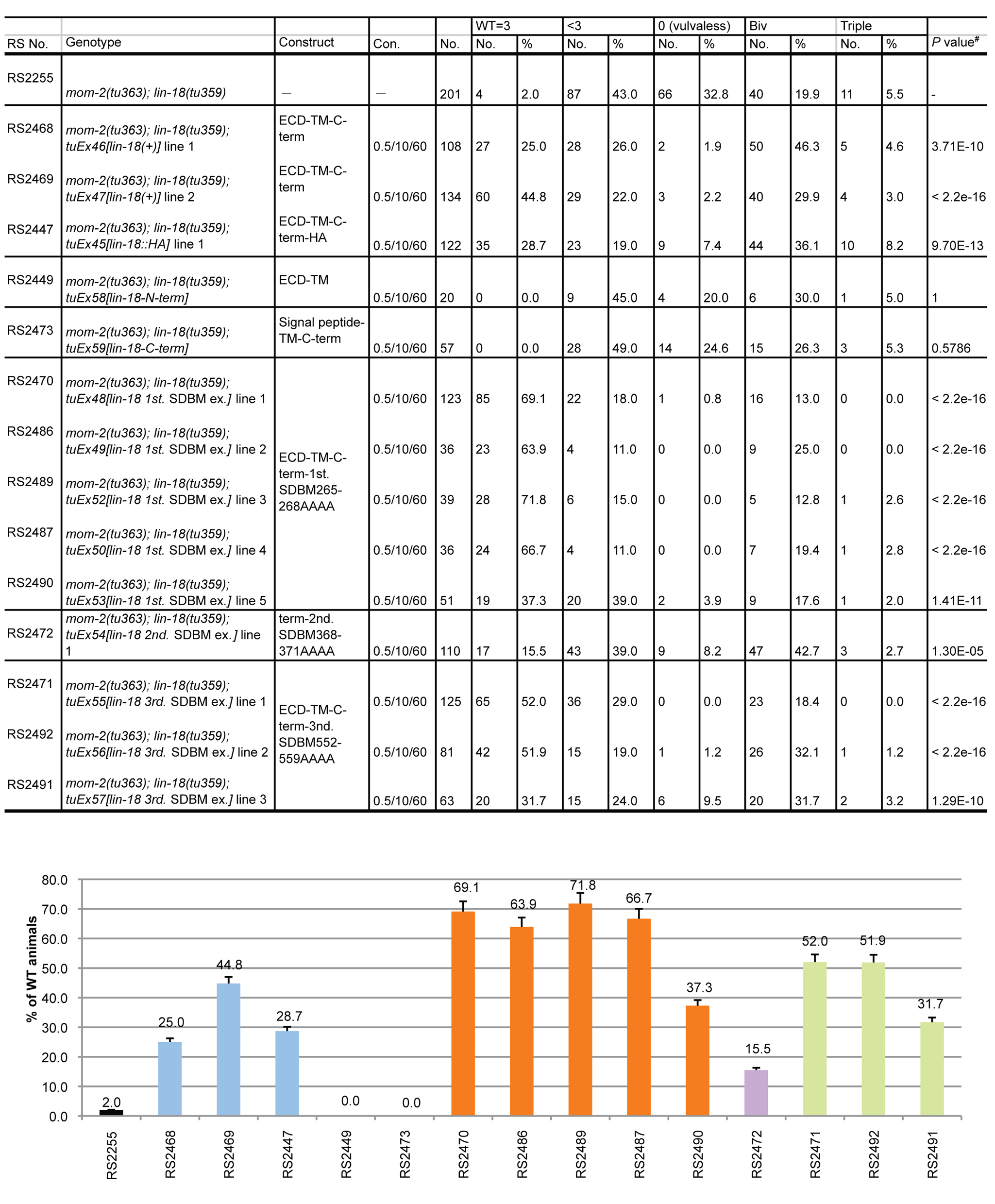

Supplement: Figure S3 — Rescue of Ppa-mom-2(tu363) ; Ppa-lin-18(tu359) by Ppa-lin-18 transgenes. Detailed description of the data summarized in Figure 3D. Late J2 or early J3 stage worms were observed. The upper part provides a description of the transgenes used in this analysis; the lower part indicates the percentage of animals with a wild-type phenotype. # p-values were calculated by comparisons with Ppa-mom-2(tu363); Ppalin-18(tu359) in percentage of wild-type animals, Fisher exact test. (TIF) [file pbio.1001110.s003.tif]

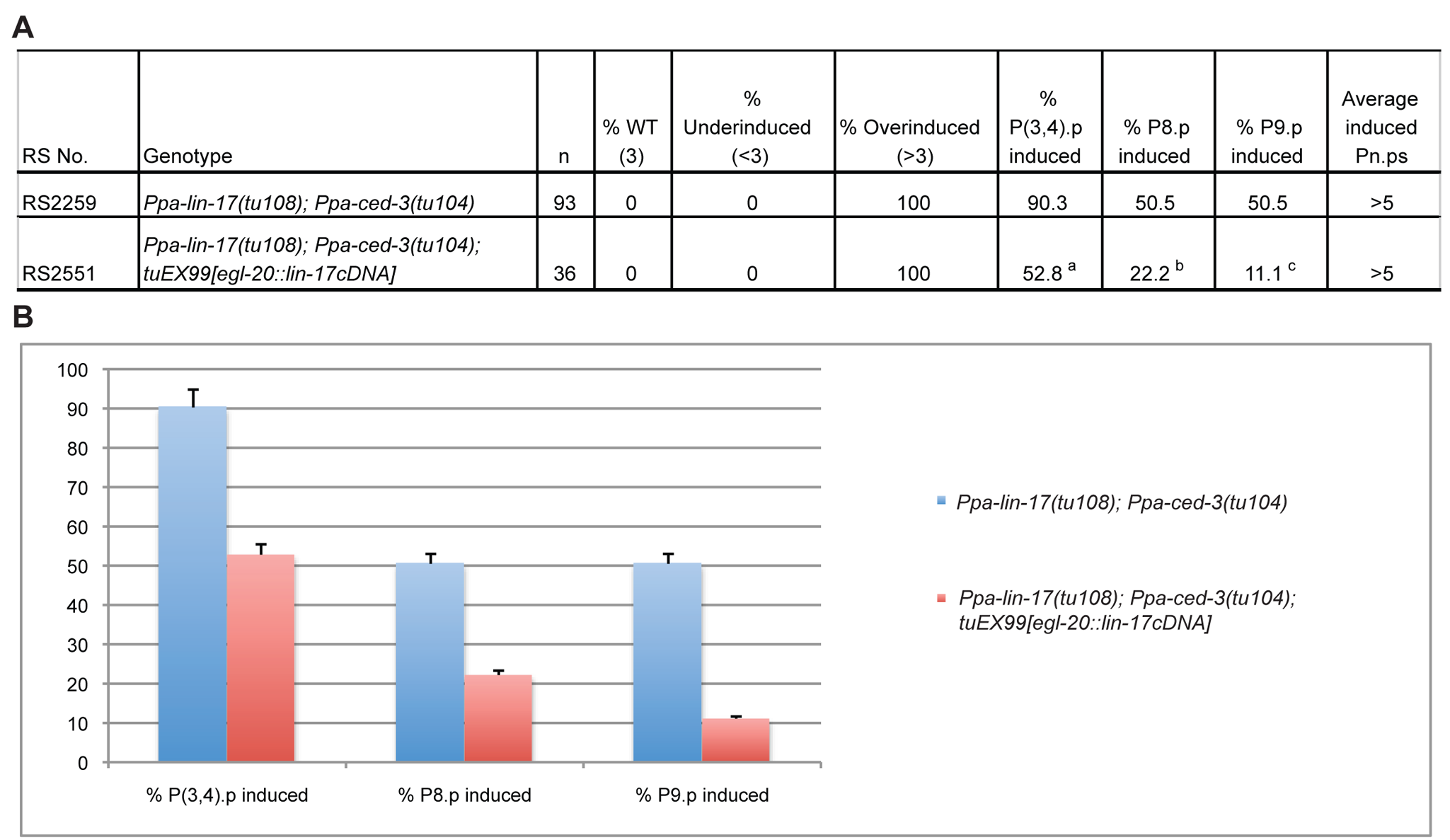

Supplement: Figure S4 — Rescue of Ppa-lin-17(tu108) multivulva phenotype by expressing LIN-17 in the tail region. (A) Late J2 or early J3 stage worms were observed. Worms with wild-type vulva induction have an average number of 3.0 induced VPCs. Statistical analysis: a p-value = 7.451e–06 [comparison with Ppa-lin-17(tu108); Ppa-ced-3(tu104) P(3,4).p vulva induction, Fisher exact test]. b p-value = 0.005066 [comparison with Ppa-lin-17(tu108); Ppa-ced-3(tu104) P8.p vulva induction]. c p-value = 3.883e–05 [comparison with Ppa-lin-17(tu108); Ppa-ced-3(tu104) P9.p vulva induction]. (B) The percentage of vulva induction in P(3,4).p, P8.p, and P9.p. The blue bars represent the percentage of vulva induction in Ppa-lin-17(tu108); Ppa-ced-3(tu104) double mutant, and the red bars represent the percentage of vulva induction in Ppa-lin-17(108); Ppa-ced-3(tu104); tuEX99[egl-20::lin-17cDNA] transgenic animals. (TIF) [file pbio.1001110.s004.tif]
