# Supplementary material for: Antagonism of LIN-17/Frizzled and LIN-18/Ryk in Nematode Vulva Induction Reveals Evolutionary Alterations in Core Developmental Pathways
Source: PLoS Biol. 2011 Jul 26;9(7):e1001110. doi: 10.1371/journal.pbio.1001110 (PMC3144188; doi:10.1371/journal.pbio.1001110)
Supplement: Table S1 — P. pacificus gene predictions for genes encoding SH3 domain containing proteins. (PDF) [file pbio.1001110.s005.pdf]

Table S1

No	*P. pacificus* gene predictions	*C. elegans* orthologs
1	Contig104-snap.89	*F44D12.1*
2	Contig11-snap.244	*F42H10.3*
3	Contig11-snap.432	*tag-168*
4	Contig120-snap.4	*ccb-1*
5	Contig12-snap.20	*tag-208*
6	Contig136-snap.27	*stam-1*
7	Contig144-snap.35	*N. A.*
8	Contig14-snap.16	*mlk-1*
9	Contig14-snap.137	*T22H9.4*
10	Contig15-snap.89	*spc-1*
11	Contig180-snap.3	*frm-2*
12	Contig18-snap.31	*toca-1*
13	Contig1-snap.232	*pix-1*
14	Contig1-snap.439	*vav-1*
15	Contig21-snap.23	*sem-5*
16	Contig22-snap.243	*eft-3*
17	Contig24-snap.212	*plc-3*
18	Contig26-snap.52	*abl-1*
19	Contig29-snap.166	*src-1*
20	Contig32-snap.135	*sem-5*
21	Contig35-snap.292	*abi-1*
22	Contig36-snap.72	*Src substrate cortactin [Brugia malayi] >gb|EDP28248.1*
23	Contig39-snap.11	*ape-1*
24	Contig3-snap.41	*hum-1*
25	Contig3-snap.121	*src-1*
26	Contig41-snap.46	*lst-4*
27	Contig448-snap.2	*ccb-1*
28	Contig44-snap.10	*Variant SH3 domain containing protein [Brugia malayi] >gb|EDP33109.1*
29	Contig44-snap.113	*dlg-1*
30	Contig46-snap.78	*B0303.7*
31	Contig48-snap.132	*itsn-1*
32	Contig4-snap.193	*tbc-18*
33	Contig50-snap.174	*sdpn-1*
34	Contig51-snap.4	*src-1*
35	Contig51-snap.40	*unc-89*
36	Contig53-snap.89	*eps-8*
37	Contig5-snap.239	*jip-1*
38	Contig61-snap.18	*ced-2*

Legend: Gene predictions of SH3 domain proteins in *P. pacificus*. The *C. elegans* orthologs are represented on the right site. In total, there are 38 predicted SH3 domain proteins in *P. pacificus*, based on the current release of the *P. pacificus* genome (www.pristionchus.org).
