# Supplementary material for: Antagonism of LIN-17/Frizzled and LIN-18/Ryk in Nematode Vulva Induction Reveals Evolutionary Alterations in Core Developmental Pathways
Source: PLoS Biol. 2011 Jul 26;9(7):e1001110. doi: 10.1371/journal.pbio.1001110 (PMC3144188; doi:10.1371/journal.pbio.1001110)
Supplement: Table S2 — Composition of the constructs used to generate transgenic lines described in this study. (PDF) [file pbio.1001110.s006.pdf]

Table S2. Transformation composition

RS No.	*tu* No.	Constructs	Marker	Genomic Carrier DNA	Conc. (ng/μl)	Genetic Background
RS2373	7	*Ppa-lin-17(1.5kb): Ppa*RFP(NLS) *rpl-23* 3'UTR (PstI)	*Ppa-egl-20*:RFP (PstI)	*ced-3(tu54)* (PstI)	10/10/60	*Ppa-ced-3(tu54)*
RS2367	33	*Dm-hsp70*::EGL-20 *rpl-23* 3'UTR (SalI)	*Ppa-egl-20*:RFP (PstI)	*Ppa-ced-3(tu104); Ppa-egl-20(tu382)* (SalI /PstI)	0.5/10/60	*Ppa-ced-3(tu104); Ppa-egl-20(tu382)*
RS2411	39	*Ppa-mig-1*::EGL-20 rpl-23 3'UTR (KpnI)		*Ppa-ced-3(tu104); Ppa-egl-20(tu382)* (KpnI/PstI)	10/10/60	*Ppa-ced-3(tu104); Ppa-egl-20(tu382)*
RS2357	16	15.7 kb genomic DNA of *Ppa-lin-17(+)* (4.1kb upstream & 2.7 kb downstream) (KpnI)	*Ppa-egl-20*:RFP (PstI)	*Ppa-lin-17(tu383)* (KpnI/PstI)	5/10/60	*Ppa-lin-17(tu383)*
RS2371	17	15.7 kb genomic DNA of *Ppa-lin-17(tu383)* (4.1kb upstream & 2.7 kb downstream) (KpnI)		*ced-3(tu54)* (KpnI/PstI)	5/10/60	*Ppa-ced-3(tu54)*
RS2434	26	*Ppa-lin-17(tu383)*PGIP574-577AAAA (KpnI)		*ced-3(tu54)* (KpnI/PstI)	5/10/60	*Ppa-ced-3(tu54)*
RS2444	27	*Ppa-lin-17(tu383)*PGIP574-577AAAA (KpnI)		*ced-3(tu54)* (KpnI/PstI)	5/10/60	*Ppa-ced-3(tu54)*
RS2436	28	*Ppa-lin-17(tu383)*THS578-580AAA (KpnI)		*ced-3(tu54)* (KpnI/PstI)	5/10/60	*Ppa-ced-3(tu54)*
RS2412	25	*Ppa-lin-17(tu383)*PGIP531-534AAAA (KpnI)		*ced-3(tu54)* (KpnI/PstI)	5/10/60	*Ppa-ced-3(tu54)*
RS2421	19	15.7 kb genomic DNA of *Ppa-lin-17(+)* (4.1kb upstream & 2.7 kb downstream) (KpnI)		*ced-3(tu54)* (KpnI/PstI)	5/10/60	*Ppa-ced-3(tu54)*
RS2523	65	LIN-17*(tu383)*PGIPTHS574-579PIGPSSSLI (KpnI)	*Ppa-egl-20*:RFP (PstI)	*ced-3(tu54)* (KpnI/PstI)	5/10/60	*Ppa-ced-3(tu54)*
RS2526	63	LIN-17*(tu383)*PGIPTHS574-579PPHPNLA (KpnI)		*ced-3(tu54)* (KpnI/PstI)	5/10/60	*Ppa-ced-3(tu54)*
RS2518	60	LIN-17*(tu383)*PGIPTHS574-579PPFPDLPS (KpnI)		*ced-3(tu54)* (KpnI/PstI)	5/10/60	*Ppa-ced-3(tu54)*
RS2441	40	6 kb genomic DNA region of *Ppa-axl-1(+)* (2.8 kb upstream & 350 bp downstream) (PstI)	*Ppa-egl-20*:RFP (PstI)	*Ppa-axl-1(tu98)* (PstI)	0.5/10/60	*Ppa-axl-1(tu98)*
RS2442						
RS2443						
RS2468	46	8 kb genomic DNA region of *Ppa-lin-18(+)* (2.5kb upstream & 700 bp downstream) (ECD-TM-C-term) (KpnI)	*Ppa-egl-20*:RFP (PstI)	*Ppa-mom-2(tu363); Ppa-lin-18(tu359)* (KpnI/PstI)	0.5/10/60	*Ppa-mom-2(tu363); Ppa-lin-18(tu359)*
RS2469	47					
RS2447	45	*Ppa-lin-18* (ECD-TM-C-term-HA) (KpnI)		*Ppa-mom-2(tu363); Ppa-lin-18(tu359)* (KpnI/PstI)	0.5/10/60	*Ppa-mom-2(tu363); Ppa-lin-18(tu359)*
RS2449	58	*Ppa-lin-18* (ECD-TM) (KpnI)		*Ppa-mom-2(tu363); Ppa-lin-18(tu359)* (KpnI/PstI)	0.5/10/60	*Ppa-mom-2(tu363); Ppa-lin-18(tu359)*
RS2473	59	*Ppa-lin-18* (Signal peptide-TM-C-term) (KpnI)		*Ppa-mom-2(tu363); Ppa-lin-18(tu359)* (KpnI/PstI)	0.5/10/60	*Ppa-mom-2(tu363); Ppa-lin-18(tu359)*
RS2470	48	*Ppa-lin-18* (ECD-TM-C-term-1st. SDBM265-268AAAA) (KpnI)		*Ppa-mom-2(tu363); Ppa-lin-18(tu359)* (KpnI/PstI)	0.5/10/60	*Ppa-mom-2(tu363); Ppa-lin-18(tu359)*
RS2486	49					
RS2489	52					
RS2487	50					
RS2490	53					
RS2472	54	*Ppa-lin-18* (ECD-TM-C-term-2nd. SDBM368-371AAAA) (KpnI)		*Ppa-mom-2(tu363); Ppa-lin-18(tu359)* (KpnI/PstI)	0.5/10/60	*Ppa-mom-2(tu363); Ppa-lin-18(tu359)*
RS2471	55	*Ppa-lin-18* (ECD-TM-C-term-3rd. SDBM552-559AAFADLAA) (KpnI)		*Ppa-mom-2(tu363); Ppa-lin-18(tu359)* (KpnI/PstI)	0.5/10/60	*Ppa-mom-2(tu363); Ppa-lin-18(tu359)*
RS2492	56					
RS2491	57					

Legend: For each microinjection mixture, the construct and the maker can be digested with different restriction enzymes, however, the carrier genomic DNA is always digested with the same restriction enzymes as both, the construct and the marker DNA. [a]Concentrations are in ng/μl and in the following order: construct, marker, and carrier genomic DNA.
